# A Predictive Model of Ischemic Heart Disease in Middle-Aged and Older Women Using Data Mining Technique

**DOI:** 10.3390/jpm13040663

**Published:** 2023-04-13

**Authors:** Jihye Lim

**Affiliations:** Department of Health Care & Science, Donga University, Busan 49315, Republic of Korea; limjiart@dau.ac.kr

**Keywords:** ischemic heart disease, data mining, decision tree, prediction, women

## Abstract

This study was conducted to identify ischemic heart disease-related factors and vulnerable groups in Korean middle-aged and older women using data from the Korea National Health and Nutrition Examination Survey (KNHANES). Among the 24,229 people who participated in the 2017–2019 survey, 7249 middle-aged women aged 40 and over were included in the final analysis. The data were analyzed using IBM SPSS and SAS Enterprise Miner by chi-squared analysis, logistic regression analysis, and decision tree analysis. The prevalence of ischemic heart disease in the study results was 2.77%, including those diagnosed with myocardial infarction or angina. The factors associated with ischemic heart disease in middle-aged and older women were identified as age, family history, hypertension, dyslipidemia, stroke, arthritis, and depression. The group most vulnerable to ischemic heart disease included women who had hypertension, a family history of ischemic heart disease, and were menopausal. Based on these results, effective management should be achieved by applying customized medical services and health management services for each relevant factor in consideration of the characteristics of the groups with potential risks. This study can be used as basic data that can be helpful in national policy decision making for the management of chronic diseases.

## 1. Introduction

Ischemic heart disease is known as a representative disease with a high social burden that causes much death and disability [1]. The prevalence of cardiovascular disease is rapidly increasing in women after the age of 40 due to changes in women’s hormones related to menopause, physical changes related to aging, and increased fat accumulation [2]. Previous studies reported that women with risk factors for cardiovascular disease had a 19% increase in the incidence of myocardial infarction after 10 years and that the quality of life of middle-aged women with cardiovascular disease was poor [3,4]. The life expectancy of Korean women in 2015 was 85.2 years [5], and the prevention and management of cardiovascular disease in middle-aged women are very important to prepare for a healthy old age. According to the 2017 Statistical Annual Report of Causes of Death in Korea, cardiovascular disease is the second-highest cause of death after cancer, and the mortality rate from cardiovascular disease also tends to increase sharply with increasing age. Particularly, hypertension disease (2.3 times) and heart disease (1.1 times) showed higher mortality rates among women than men [6]. Cardiovascular disease is a major chronic disease. Chronic diseases have various causes but no direct cause, making early diagnosis difficult. In addition, the time of disease onset is unclear and the latent period is long [7]. Therefore, since prevention is emphasized more than treatment in chronic diseases, if the characteristics of a group at high risk of cardiovascular disease can be identified and customized interventions suitable for each characteristic can be provided, the prevention and management of cardiovascular disease will be effective.

The risk factors for cardiovascular disease identified in previous studies included gender, age, marital status, income, education, diabetes, hypercholesterolemia, family history, smoking, drinking, obesity, lack of physical activity, and stress [8,9,10,11,12,13,14,15,16,17,18,19]. However, these studies investigated the incidence of cardiovascular disease related to a few factors by focusing on specific groups such as young men and the elderly as study subjects [8,9,12,13,14,15,16,18,19]. Studies that have confirmed various characteristics in the groups vulnerable to cardiovascular disease are lacking. Therefore, to understand the characteristics of the groups vulnerable to ischemic heart disease, research using data mining techniques is required.

Data mining technology allows for the exploration, identification, and modeling of the relationships and rules that exist in big data [20]. Recently, research methods using data mining have been used in various fields such as medical research, diagnosis, quality control, hospital management, and customer relationship management in the medical field [21,22,23]. Decision tree analysis, one data mining technique, is an effective tool for classification and prediction; therefore, it is useful for discovering hidden patterns in data [24]. Predicting cardiovascular disease risk using decision support systems can play an important role in disease prevention [24].

In this study, we intended to analyze the factors related to ischemic heart disease in middle-aged women using the Korea National Health and Nutrition Examination Survey (KNHANES) data, which is representative of the Korean middle-aged and older women population, and develop an ischemic heart disease prediction model. The specific study purposes were as follows.

- Identify the sociodemographic characteristics and health-related behavior characteristics of the study subjects.
- Identify the differences in the prevalence of ischemic heart disease according to social demographic characteristics and health-related behaviors, and the presence of chronic diseases.
- Identify the factors that affected ischemic heart disease in middle-aged women.
- Utilizing data mining techniques, develop a predictive model for ischemic heart disease in middle-aged women.

The results of these studies can be used as important foundational data for regional and national health policy decisions for the prevention and management of ischemic heart disease.

## 2. Materials and Methods

### 2.1. Study Population

**Figure 1:**
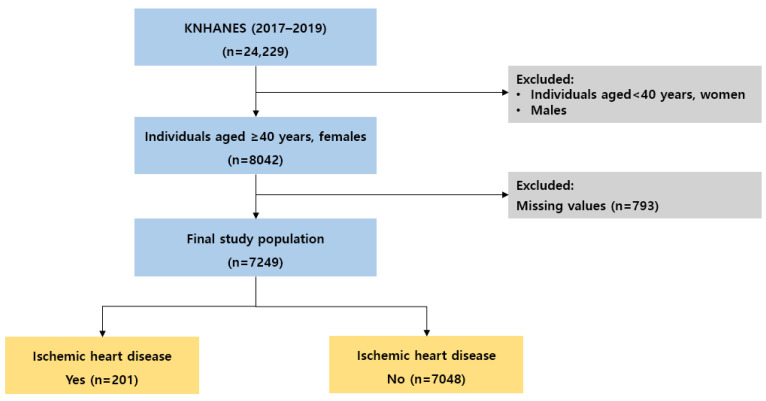
Study population selection process.

In this study, the raw data of the Korea National Health and Nutrition Examination Survey (KNHANES) (2017–2019), which was a statutory survey based on Article 16 of the National Health Promotion Law, were utilized. Data use was approved according to the procedures of the Korea Disease Control and Prevention Agency (KDCA). The target population of the KNHANES was one year old or older residing in Korea, and a two-step stratified cluster sampling method using the survey district and household as the primary and secondary extraction units, respectively, was applied. The number of survey districts was 192 and there were 23 sampled households. Within the sampled households, all household members aged one year or older who satisfied the appropriate household size were selected as survey subjects. Among the 24,229 people who participated in the 2017–2019 survey, 7249 middle-aged women aged 40 and over were included in the final analysis after excluding missing data (Figure 1). The age groups of the study subjects were 40–49 years old (1822 people), 50–59 years old (1966 people), 60–69 years old (1761 people), 70–79 years old (1257 people), and 80 years old and over (443 people).

The study was conducted in accordance with the Declaration of Helsinki. Ethical review and approval were waived for this study because it used anonymous public open data and not an individual’s personal data.

### 2.2. Variable Definitions

The dependent variable for the presence or absence of ischemic heart disease utilized the answer to the question “Have you ever been diagnosed with myocardial infarction or angina by your doctor?” The characteristics of the study subjects were classified into sociodemographic factors, health behavior factors, and clinical factors. The independent variables for sociodemographic characteristics were age, marital status, education, household income, subjective health status, and stress. Marital status was divided into married and unmarried, and education levels were classified as less than elementary school, middle school, high school, and college or higher. Household income levels were divided into categories (lower, lower middle, upper middle, and upper) based on the quartile of household equalization income. The independent variables for health behavior characteristics were smoking, alcohol drinking, and physical activity. Smoking status was divided into daily smoking, occasionally smoking, past smoker, and non-smoker. Drinking was divided according to the classification of the raw data, with and without the experience of drinking alcohol for a lifetime. The physical activity variable used the response to the question “Does your work or leisure activity involve moderate-intensity physical activity with a slight shortness of breath or moderately rapid heart rate for at least 10 min?” The clinical characteristic variables were composed of body mass index (BMI), menopause, family history, hypertension, stroke, arthritis, diabetes mellitus, depression, renal failure, and dyslipidemia, with reference to previous studies [7,10,24,25,26]. BMI was classified as underweight for a value of less than 18.5 kg/m^2^, normal for 18.5 kg/m^2^ or more and less than 25.0 kg/m^2^, and obesity for 25.0 kg/m^2^ or more. Family history was defined as when at least one parent or sibling had a history of ischemic heart disease.

### 2.3. Statistical Analysis

The data were analyzed using IBM SPSS version 25.0 (IBM Co., Armonk, NY, USA) and SAS Enterprise Miner 9.4. To observe the differences in the prevalence of ischemic heart disease according to social demographic characteristics, health-related behaviors, and the presence of chronic diseases, a chi-squared analysis was conducted. Logistic regression analysis was performed to identify the factors influencing the prevalence of ischemic heart disease. The statistical significance level was set as a two-sided test of *p* < 0.05. An interactive decision tree analysis and random forest analysis were generated to develop a predictive model of ischemic heart disease.

## 3. Results

### 3.1. General Characteristics of the Study Regions

The distribution of ischemic heart disease cases according to the general characteristics is described in Table 1. The prevalence of ischemic heart disease was high among women aged 80 years (7.7%) and over and those with a low educational attainment of less than elementary school (5.6%) (Table 1). It was found that the presence of ischemic heart disease was high in women with low household incomes (5.3%), women who experienced very poor subjective health (9.1%), and women who experienced a lot of stress (4.8%) (Table 1). The prevalence of ischemic heart disease according to age, education level, household income, subjective health status, and stress awareness was statistically significantly different (*p* < 0.05) (Table 1).

### 3.2. Health Behavior and Clinical Characteristics of the Study Regions

The prevalence of ischemic heart disease according to health behavior characteristics is shown in Table 2. Among the variables of smoking, drinking, and physical activity, there was a statistically significant difference only in the prevalence of ischemic heart disease with or without drinking experience (Table 2).

The distribution of ischemic heart disease cases according to the clinical characteristics is described in Table 3. The presence of ischemic heart disease was high among obese (4.1%) and menopausal women (3.8%), and those with a family history of ischemic heart disease (5.0%) (Table 3). Moreover, the prevalence of ischemic heart disease was statistically significantly higher if there were comorbidities including hypertension, dyslipidemia, stroke, arthritis, diabetes mellitus, depression, and renal failure (*p* < 0.05) (Table 3).

### 3.3. Predictive Factors of Ischemic Heart Disease

Logistic regression analysis was performed to identify the factors related to ischemic heart disease in middle-aged women (Table 4). The analysis showed that ischemic heart disease in middle-aged women was significantly associated with age, physical leisure activity, family history, hypertension, dyslipidemia, stroke, arthritis, and depression (Table 4). The incidence of ischemic heart disease was 16.73 times higher in people over 80 years old than in those 40–49 years old. The incidence of ischemic heart disease in those with a family history was 3.29 times (95% confidence interval (CI): 2.03–5.32) higher than in those without a family history, and 1.42 times (95% CI: 1.01–2.00) higher in patients with hypertension than in those without hypertension (Table 4). The risk of ischemic heart disease was more than 1.70 times (95% CI: 1.24–2.33) higher in patients with dyslipidemia and more than 1.93 times (95% CI: 1.18–3.18) higher in those with a previous stroke. The risk of ischemic heart disease was more than 1.43 times (95% CI: 1.05–1.94) higher in patients with arthritis and more than 1.66 times (95% CI 1.09–2.51) higher in patients with depression (Table 4).

**Figure 2:**
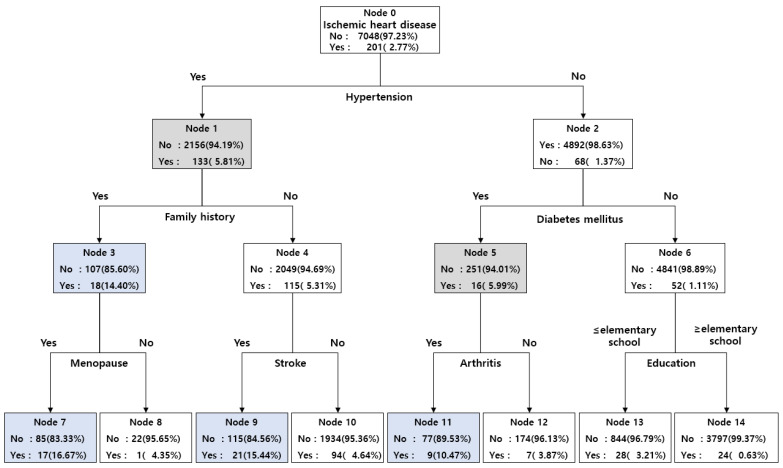
Prediction of ischemic heart disease in middle-aged and older women using a decision tree.

Decision tree analysis was performed to identify the ischemic heart disease risk group in the study subjects. As for the method of growing the trees, the classification and regression tree (CRT) method was applied to maximize homogeneity within the child nodes by separating them to be as homogeneous as possible within the child nodes (Figure 2). At the researcher’s discretion, we presented an interactive decision tree analysis focusing on health behavior and clinical characteristics, excluding age variables that were too strongly associated in logistic regression analysis. As a result of the analysis, a total of eight nodes were separated based on the terminal node, and the seventh node (16.67%) was found to be the most vulnerable to ischemic heart disease (Figure 2). The seventh node was a patient with hypertension, a family history of ischemic heart disease, and menopause. The group with hypertension had a higher risk of developing ischemic heart disease than the group without hypertension, and the group without hypertension had the highest risk of developing ischemic heart disease at the eleventh node at 10.47% in patients with diabetes and arthritis (Figure 2).

An interactive decision tree analysis and random forest analysis were generated to develop a predictive model of ischemic heart disease. The results of the random forest algorithm analyzed to predict the presence or absence of ischemic heart disease are shown in Table 5. As a result of the random forest analysis, the important variables in predicting ischemic heart disease response were age, dyslipidemia, education level, arthritis, hypertension, diabetes, depression, family history, menopause, and stroke, in that order (Table 5). For modeling comparison, logistic regression, decision trees, and random forest algorithms were used to compare prediction models for each dependent variable. The sensitivity, specificity, and accuracy of each model were confirmed, and the model was evaluated using AUC. For the AUC value, the closer the area of the ROC curve is to 1, the better the performance of the model. If the AUC value is 0.8 or more, it is evaluated as a stable model, and the AUC values of all three prediction models presented in this study showed 0.8 or more. The AUC value has the highest value at 0.872 in random forest. All of the models’ accuracy, sensitivity, and specificity showed the highest values in random forest (Table 6).

## 4. Discussion

With the development of medical technology, life expectancy has increased, and women spend more than a third of their lives after middle age. The middle-aged period of women is the beginning period of before and just after the onset of menopause, and since health management after middle-age is closely related to the quality of life, active health management is necessary [27]. Therefore, this study was performed to contribute to the prevention and management of ischemic heart disease for health promotion by identifying the factors related to ischemic heart disease in middle-aged and older Korean women and identifying the vulnerable group with a high prevalence of ischemic heart disease.

The prevalence of ischemic heart disease in the study was 2.77%, including those diagnosed with myocardial infarction or angina. It was slightly higher than the results of previous studies [28], which suggested that about 1.72% of the world’s population is affected by ischemic heart disease. When the prevalence of ischemic heart disease was compared by age, it increased rapidly after 60 years old compared to those 40–49 years old. Previous studies have also shown that cardiovascular disease increased rapidly after 50 years old [29,30]. In particular, it is known that as women transition from middle age to old age, the incidence of cardiovascular disease increases due to changes in women hormones, physical changes according to aging, and an increase in body fat accumulation [2,27].

In this study, family history, hypertension, dyslipidemia, stroke, arthritis, and depression were found to be statistically significant as clinical factors affecting ischemic heart disease, and smoking, drinking, and physical activity were not related factors. Since the association was investigated in middle-aged and older women, the results differed from previous studies [9,11,29,31,32] where smoking, drinking, and physical activity were associated with ischemic heart disease. Previous studies were conducted on both men and women with cardiovascular disease [9] and on women in their 30s or older [11], and it is thought that the results were different because they were more than the data set of cardiovascular disease patients used in this study. According to a previous study by Lim [33] using machine learning, the major risk factors affecting the occurrence of myocardial infarction and angina were age, hypertension, dyslipidemia, family history, low educational background, and gender, consistent with the results of this study. The diseases identified as risk factors for cardiovascular in this study were hypertension, dyslipidemia, stroke, arthritis, and depression. However, since it is difficult to clearly identify a causal relationship in a cross-sectional study, it is also possible that individuals with ischemic heart disease may have had high prevalence of risk factors for comorbidities such as hypertension and dyslipidemia due to more frequent health care encounters and screening opportunities. Diabetes and renal failure did not show a statistically significant association. These results were similar to previous studies [12,14,26,34,35] reporting that the cardiovascular disease risk factors depression and rheumatoid arthritis were significantly higher in women than men, and diabetes was statistically significantly higher in men. In a study by Seo et al. [36], Korean adults with depression had a higher prevalence of cardiovascular disease than those without depression, and a previous study confirmed depression as a significant cardiovascular disease risk factor in women compared to men [35]. Decreased renal function may increase the prevalence of cardiovascular disease and increase mortality [37]. However, in this study, it was not a risk factor in middle-aged and older women.

As a result of the decision tree analysis to identify the groups vulnerable to ischemic heart disease, hypertension and family history were derived as the most relevant factors, consistent with the regression analysis. Focusing on hypertension, which is the biggest influencing factor, those who had hypertension, a family history, and were menopausal (16.67%), and those who had hypertension, no family history, and had a previous stroke (15.44%) were found to be the groups most vulnerable to ischemic heart disease. Taken together, the risk of ischemic heart disease increased in middle-aged and older women when combined with related factors such as hypertension, a family history of ischemic heart disease, menopause, and stroke. These results are consistent with the results of previous studies [38,39,40] that postmenopausal women significantly increase the risk of cardiovascular disease.

The study results indicated that for the prevention and effective management of ischemic heart disease in middle-aged and older women, a customized program considering the characteristics of the subjects is intensively needed. The importance of women’s health care after middle age is emphasized, but in most cases, a uniform program is applied by integrating factors affecting cardiovascular disease [7]. In previous studies [8,9,10,11,12,13,14,15,16,17,18,19], risk factors for ischemic heart disease were selected based on socioeconomic characteristics, some co-morbidities, and clinical test results. However, in this study, most of the comorbidities, socioeconomic characteristics, and lifestyle behaviors suggested to be related in previous studies were reflected and analyzed. In addition, there is a lack of previous studies that have identified factors affecting ischemic heart disease and risk groups in middle-aged women. According to the results of this study, family history, vascular disease, and depression appeared to be the biggest risk factors for cardiovascular disease in middle-aged women, rather than menopause and lifestyle, which can be seen as a different result from previous studies. Based on the results of this study, for the prevention and management of ischemic heart disease in middle-aged and older women, it is necessary to first classify the subjects according to the risk level of each vulnerable group. In addition, it is necessary to establish a customized prevention and management strategy according to the characteristics of the relevant factors in each vulnerable group. Utilization of healthcare big data can contribute to enormous cost savings in the healthcare field by providing patient-customized medical services based on data. E-health and m-health devices that combine technologies such as big data, data mining, and deep learning are bringing about innovation in the medical field, such as disease prevention, diagnosis, and treatment, by providing more effective and personalized solutions. If we add the function of identifying and managing high-risk and low-risk patients in advance using a predictive model to this technological system, we believe that it can contribute to the management of ischemic heart disease in middle-aged women. This study is significant in that it identified the characteristics of middle-aged and older women who are vulnerable to ischemic heart disease using large-scale data representing the entire Korean population. However, it had the following limitations. First, this study was cross-sectional, making the investigation of the cause–effect relationship between the risk factors for ischemic heart disease difficult. Second, there was a lack of data on clinical examinations related to ischemic heart disease. Third, since the ischemic heart disease variables used in this study were obtained as self-reported data on doctors’ diagnoses, there is a limitation that there may be a bias toward memory recall. In the future, it is necessary to analyze big data mining techniques in more depth with more data and conduct a prospective cohort study on the relationship between risk factors for ischemic heart disease by addressing the limitations of this study.

## 5. Conclusions

This study was conducted to identify ischemic heart disease-related factors and the vulnerable groups in Korean middle-aged and older women using data from the Korea National Health and Nutrition Examination Survey (KNHANES). The factors associated with ischemic heart disease in middle-aged and older women in this study were age, family history, hypertension, dyslipidemia, stroke, arthritis, and depression. Additionally, the group most vulnerable to ischemic heart disease were those with high blood pressure, a family history of ischemic heart disease, and menopause. It is meaningful that research to develop a predictive model for ischemic heart disease, with a high social burden of disease, using healthcare big data can be used as basic data to help in national policy decision-making for the prevention and management of chronic diseases. Based on these results, effective management should be achieved by applying customized medical services and health management services for each relevant factor in consideration of the characteristics of the groups with potential risk. In addition, it is necessary to reflect the realization of active screening programs and chronic disease management education programs that consider the comorbidity of patients with ischemic heart disease in health policies.

## Figures and Tables

**Table 1:** Presence of ischemic heart disease according to general characteristics.

Variables	Ischemic Heart Disease		Total (*n* = 7249)	*p*-Value ^1^
	Yes (*n*, %)	No (*n*, %)		
Age				
40–49	3 (0.2)	1819 (99.8)	1822	<0.000
50–59	17 (0.9)	1949 (99.1)	1966	
60–69	62 (3.5)	1699 (96.5)	1761	
70–79	85 (6.8)	1172 (93.2)	1257	
≥80	34 (7.7)	409 (92.3)	443	
Marital status				
Yes	198 (2.8)	6871 (97.2)	7069	0.360
No	3 (1.7)	177 (98.3)	180	
Education level				
≤Elementary school	133 (5.6)	2221 (94.4)	2534	<0.000
Middle school	31 (3.4)	875 (96.6)	906	
High school	27 (1.2)	2179 (98.8)	2206	
≥College	10 (0.6)	1773 (99.4)	1783	
Household income level				
Lower	97 (5.3)	1737 (94.7)	1834	<0.000
Lower middle	41 (2.3)	1747 (97.7)	1788	
Upper middle	43 (2.5)	1695 (97.5)	1738	
Upper	20 (1.1)	1869 (98.9)	1889	
Subjective health status				
Very good	1 (0.4)	241 (99.6)	242	<0.000
Good	13 (0.9)	1427 (99.1)	1440	
Usually	81 (2.1)	3776 (97.9)	3857	
Poor	66 (5.2)	1203 (94.8)	1269	
Very poor	40 (9.1)	401 (90.9)	441	
Stress awareness				
Very much	16 (4.8)	315 (95.2)	331	<0.000
A lot	49 (3.3)	1426 (96.7)	1475	
A little bit	85 (2.1)	3989 (97.9)	4074	
Rarely	51 (3.7)	1318 (96.3)	1369	

^1^ *p*-value by chi-square test. *p* < 0.05.

**Table 2:** Presence of ischemic heart disease according to health behavior.

Variables	Ischemic Heart Disease		Total (*n* = 7249)	*p*-Value ^1^
	Yes (*n*, %)	No (*n*, %)		
Smoking				
Daily smoking	8 (3.7)	210 (96.3)	218	0.775
Occasionally smoking	2 (2.8)	69 (97.2)	71	
Past smoker	12 (3.3)	352 (96.7)	364	
Non-smoker	179 (2.7)	6417 (96.7)	6596	
Drinking				
Yes	139 (2.4)	5661 (97.6)	5800	<0.000
No	62 (4.3)	1387 (95.7)	1449	
Moderate physical activity (work)				
Yes	9 (3.3)	263 (96.7)	272	0.583
No	192 (2.8)	6785 (97.2)	6977	
Moderate physical activity (leisure)				
Yes	28 (2.2)	1252 (97.8)	1280	0.160
No	173 (2.9)	5796 (97.1)	5969	

^1^ *p*-value by chi-square test. *p* < 0.05.

**Table 3:** Presence of ischemic heart disease according to clinical characteristics.

Variables	Ischemic Heart Disease		Total (*n* = 7249)	*p*-Value ^1^
	Yes (*n*, %)	No (*n*, %)		
BMI				
Underweight	3 (1.4)	205 (98.6)	208	<0.000
Normal weight	99 (2.1)	4537 (97.9)	4636	
Obesity	99 (4.1)	2306 (95.9)	2405	
Menopause				
Yes	192 (3.8)	4801 (96.2)	4993	<0.000
No	9 (0.4)	2247 (99.6)	2256	
Family history				
Yes	25 (5.0)	479 (95.0)	504	0.002
No	176 (2.6)	6569 (97.4)	6745	
Hypertension				
Yes	133 (5.8)	2156 (94.2)	2289	<0.000
No	68 (1.4)	4892 (98.6)	4960	
Dyslipidemia				
Yes	119 (5.8)	1932 (94.2)	2051	<0.000
No	82 (1.6)	5116 (98.4)	5198	
Stroke				
Yes	23 (11.7)	173 (88.3)	196	<0.000
No	178 (2.5)	6875 (97.5)	7053	
Arthritis				
Yes	112 (6.0)	1743 (94.0)	1855	<0.000
No	89 (1.6)	5305 (98.4)	5394	
Diabetes mellitus				
Yes	56 (6.9)	757 (93.1)	813	<0.000
No	145 (2.3)	6291 (97.7)	6436	
Depression				
Yes	34 (6.7)	470 (93.3)	504	<0.000
No	167 (2.5)	6578 (97.5)	6745	
Kidney failure				
Yes	3 (11.5)	23 (88.5)	26	0.006
No	198 (2.1)	7025 (97.3)	7223	

^1^ *p*-value by chi-square test. *p* < 0.05.

**Table 4:** Predictive factors for the presence of ischemic heart disease.

Variables	Categories	Ischemic Heart Disease	
		OR	95% CI
Age	40–49	1	
	50–59	2.83	0.71–11.25
	60–69	7.51 *	1.87–30.23
	70–79	13.82 *	3.34–57.25
	≥80	16.73 *	3.86–72.46
Marital status	Yes	1	
	No	1.22	0.35–4.20
Education level	≤Elementary school	1	
	Middle school	1.03	0.66–1.59
	High school	0.75	0.46–1.24
	≥College	0.58	0.28–1.22
Household income level	Upper	1	
	Upper middle	0.92	0.53–1.62
	Lower middle	0.81	0.45–1.45
	Lower	1.49	0.85–2.62
Subjective health status	Very good	1	
	Good	2.49	0.32–19.36
	Usually	3.81	0.52–27.94
	Poor	6.56	0.89–48.44
	Very poor	6.89	0.91–51.95
Stress awareness	Rarely	1	
	A little bit	1.19	0.63–2.24
	A lot	1.13	0.73–1.76
	Very much	0.89	0.61–1.31
Smoking	Non-smoker	1	
	Past smoker	1.85	0.85–4.01
	Occasionally smoking	2.40	0.52–11.03
	Daily smoking	1.55	0.82–2.93
Drinking	No	1	
	Yes	1.11	0.80–1.54
Moderate physical activity (work)	Yes	1	
	No	0.84	0.41–1.73
Moderate physical activity (leisure)	Yes	1	
	No	0.63 *	0.41–0.99
BMI	Underweight	1	
	Normal weight	1.08	0.33–3.58
	Obesity	1.43	0.43–4.77
Menopause	No	1	
	Yes	1.46	0.65–3.28
Family history	No	1	
	Yes	3.29 *	2.03–5.32
Hypertension	No	1	
	Yes	1.42 *	1.01–2.00
Dyslipidemia	No	1	
	Yes	1.70 *	1.24–2.33
Stroke	No	1	
	Yes	1.93 *	1.18–3.18
Arthritis	No	1	
	Yes	1.43 *	1.05–1.94
Diabetes mellitus	No	1	
	Yes	1.27	0.90–1.79
Depression	No	1	
	Yes	1.66 *	1.09–2.51
Kidney failure	No	1	
	Yes	1.86	0.52–6.70

Logistic regression analysis: classification accuracy, 97.2%, * *p* < 0.05; OR: odds ratio, CI: confidence interval.

**Table 5:** Prediction of ischemic heart disease in middle-aged and older women using a random forest.

Variables	Gini Importance	Gini Importance (Out of Bagging)
Age	0.012	0.013
Dyslipidemia	0.009	0.009
Education level	0.008	0.007
Arthritis	0.007	0.006
Hypertension	0.007	0.006
Diabetes mellitus	0.006	0.004
Depression	0.006	0.004
Family history	0.006	0.003
Menopause	0.005	0.003
Stroke	0.004	0.002

**Table 6:** Model evaluation.

	Logistic Regression	Decision Tree	Random Forest
Sensitivity	0.214	0.199	0.433
Specificity	0.982	0.977	0.992
Accuracy	0.861	0.856	0.877
AUC (Area Under Curve)	0.852	0.848	0.872

## Data Availability

Restrictions apply to the availability of these data. Data were obtained from the Korea Disease Control and Prevention Agency (KDCA) and are available from https://knhanes.kdca.go.kr/knhanes/sub03/sub03_02_05.do (accessed on 1 August 2021).

## References

[1] Sezavar S., Valizade M., Moradi M., Rahbar M. (2010). Review of early myocardial infarction and its risk factor in patients hospitalized in Rasool Akram hospital in Tehran. Hormozgan Med. J..

[2] Yeoum S. (2003). The investigation on the risk factors of cardiovascular disease for postmenopausal women over 50 years. J. Korean Soc. Menopause.

[3] Mosca L., Benjamin E.J., Berra K., Bezanson J.L., Dolor R.J., Lloyd-Jones D.M., Newby L.K., Pina I.L., Roger V.L., Shaw L.J. (2011). Effectiveness-based guidelines for the prevention of cardiovascular disease in women—2011 update: A guideline from the American Heart Association. Circulation.

[4] Ludt S., Wensing M., Szecsenyi J., Van Lieshout J., Rochon J., Freund T., Campbell S.M., Ose D. (2011). Predictors of health-related quality of life in patients at risk for cardiovascular disease in European primary care. PLoS ONE.

[5] Korea S. (2017). The Lives of Women Looking to 2017 Statistics [Internet].

[6] Korea S. (2020). Causes of Death Statistics [Internet].

[7] Kang M.J., Yi J.S., Park C.S. (2018). Factors related to the identification of middle-aged women who are disadvantaged by cardio-cerebrovascular disease. Korean J. Women Health Nurs..

[8] Kim C.-G., Lee S.H., Cha S.K. (2017). Influencing factors on cardio-cerebrovascular disease risk factors in young men: Focusing on obesity indices. J. Korean Biol. Nurs. Sci..

[9] Hong S., Byeon H., Kim J., Mun S. (2015). Development of risk prediction model for cardiovascular disease among community-dwelling elderly. Asia-Pac. J. Multimid. Serv. Converg. Art Hum. Soc..

[10] Choi J.-Y., Choi S.-W. (2014). Comparison of the health behaviors according to income and education level among cardio-cerebrovascular patients; based on KNHANES data of 2010–2011. J. Korea Acad.-Ind. Coop. Soc..

[11] Park K.J., Lim G.U., Hwangbo Y., Jhang W.G. (2011). The impact of health behaviors and social strata on the prevalence of cardio-cerebrovascular disease. Soonchunhyang Med. Sci..

[12] Barr E.L., Zimmet P.Z., Welborn T.A., Jolley D., Magliano D.J., Dunstan D.W., Cameron A.J., Dwyer T., Taylor H.R., Tonkin A.M. (2007). Risk of cardiovascular and all-cause mortality in individuals with diabetes mellitus, impaired fasting glucose, and impaired glucose tolerance: The Australian Diabetes, Obesity, and Lifestyle Study (AusDiab). Circulation.

[13] Cordero A., Andrés E., Ordoñez B., León M., Laclaustra M., Grima A., Luengo E., Moreno J., Bes M., Pascual I. (2009). Usefulness of triglycerides-to–high-density lipoprotein cholesterol ratio for predicting the first coronary event in men. Am. J. Cardiol..

[14] Fried L.F., Shlipak M.G., Crump C., Kronmal R.A., Bleyer A.J., Gottdiener J.S., Kuller L.H., Newman A.B. (2003). Renal insufficiency as a predictor of cardiovascular outcomes and mortality in elderly individuals. J. Am. Coll. Cardiol..

[15] He Y., Jiang B., Wang J., Feng K., Chang Q., Zhu S., Fan L., Li X., Hu F.B. (2007). BMI versus the metabolic syndrome in relation to cardiovascular risk in elderly Chinese individuals. Diabetes Care.

[16] Wong N.D., Lopez V.A., Roberts C.S., Solomon H.A., Burke G.L., Kuller L., Tracy R., Yanez D., Psaty B.M. (2010). Combined association of lipids and blood pressure in relation to incident cardiovascular disease in the elderly: The cardiovascular health study. Am. J. Hypertens..

[17] Yun J.W., Lee W.Y., Kim J.Y., Park H.D., Lim S.H., Jung C.H., Kim Y.C., Kim S.W. (2002). Relationship between body fat distribution and atherosclerotic risk factors in Korean populations. Korean J. Med..

[18] Yoo S.Y., Kim M., Kim S., Kim S.H., Ko S.J., Beom J.W., Kim J.Y., Jo J., Kim Y.U., Heo D. (2013). Relationship between obesity indices and cardiovascular risk score in Korean type 2 diabetes patients. Korean J. Obes..

[19] Steptoe A., Kivimäki M. (2012). Stress and cardiovascular disease. Nat. Rev. Cardiol..

[20] Fayyad U.M., Piatetsky-Shapiro G., Smyth P. (1996). Knowledge Discovery and Data Mining: Towards a Unifying Framework. In KDD.

[21] Islam M.S., Hasan M.M., Wang X., Germack H.D. (2018). A systematic review on healthcare analytics: Application and theoretical perspective of data mining. Healthcare.

[22] Kumar V., Mishra B.K., Mazzara M., Thanh D.N., Verma A. (2020). Prediction of malignant and benign breast cancer: A data mining approach in healthcare applications. Advances in Data Science and Management.

[23] Park M., Choi S., Shin A.M., Koo C.H. (2013). Analysis of the characteristics of the older adults with depression using data mining decision tree analysis. J. Korean Acad. Nurs..

[24] Safdari R., Saeedi M.G., Arji G., Gharooni M., Soraki M., Nasiri M. (2013). A model for predicting myocardial infarction using data mining techniques. Front. Health Inform..

[25] Dosi R., Bhatt N., Shah P., Patell R. (2014). Cardiovascular disease and menopause. J. Clin. Diagn. Res..

[26] Oh M.S., Jeong M.H. (2020). Sex differences in cardiovascular disease risk factors among Korean adults. Korean J. Med..

[27] Lowdermilk D.L., Cashion M.C., Perry S.E., Alden K.R., Olshansky E. (2019). Maternity and Women’s Health Care E-Book.

[28] Khan M.A., Hashim M.J., Mustafa H., Baniyas M.Y., Al Suwaidi S.K.B.M., AlKatheeri R., Alblooshi F.M.K., Almatrooshi M.E.A.H., Alzaabi M.E.H., Al Darmaki R.S. (2020). Global epidemiology of ischemic heart disease: Results from the global burden of disease study. Cureus.

[29] Bae Y., Lee K. (2016). Risk factors for cardiovascular disease in adults aged 30 years and older. J. Korean Soc. Integr. Med..

[30] Joo J.K., Son J.B., Jung J.E., Kim S.C., Lee K.S. (2012). Differences of prevalence and components of metabolic syndrome according to menopausal status. J. Korean Soc. Menopause.

[31] Moon H.-K., Kong J.-E. (2010). Assessment of nutrient intake for middle aged with and without metabolic syndrome using 2005 and 2007 Korean National Health and Nutrition Survey. Korean J. Nutr..

[32] Kim S. (2016). Incidence Rate and Risk Factors of Cardio-Cerebrovascular Disease of Middle-Aged and Elderly People. Master’s Thesis.

[33] Lim H.K. (2018). Prediction of myocardial infarction/angina and selection of major risk factors using machine learning. J. Korean Data Anal. Soc..

[34] Turner R., Millns H., Neil H., Stratton I., Manley S., Matthews D., Holman R. (1998). Risk factors for coronary artery disease in non-insulin dependent diabetes mellitus: United Kingdom Prospective Diabetes Study (UKPDS: 23). BMJ.

[35] Jung Y.H., Shin H.K., Kim Y.H., Shin H.G., Linton J. (2017). The association of depression and cardiovascular risk factors in Korean adults: The sixth Korea National Health and Nutrition Examination Survey, 2014. Korean J. Fam. Pract..

[36] Seo Y., Je Y. (2018). A comparative study on cardiovascular disease risk factors in Korean adults according to clinical depression status. Psychiatry Res..

[37] Bae E.H., Lim S.Y., Cho K.H., Choi J.S., Kim C.S., Park J.W., Ma S.K., Jeong M.H., Kim S.W. (2012). GFR and cardiovascular outcomes after acute myocardial infarction: Results from the Korea Acute Myocardial Infarction Registry. Am. J. Kidney Dis..

[38] El Khoudary S.R., Aggarwal B., Beckie T.M., Hodis H.N., Johnson A.E., Langer R.D., Limacher M.C., Manson J.E., Stefanick M.L., Allison M.A. (2020). Menopause transition and cardiovascular disease risk: Implications for timing of early prevention: A scientific statement from the American Heart Association. Circulation.

[39] Zhu D., Chung H.-F., Dobson A.J., Pandeya N., Giles G.G., Bruinsma F., Brunner E.J., Kuh D., Hardy R., Avis N.E. (2019). Age at natural menopause and risk of incident cardiovascular disease: A pooled analysis of individual patient data. Lancet Public Health.

[40] Nappi R.E., Simoncini T. (2021). Menopause transition: A golden age to prevent cardiovascular disease. Lancet Diabetes Endocrinol..

